# Entropy Generation Rates in Two-Dimensional Rayleigh–Taylor Turbulence Mixing

**DOI:** 10.3390/e20100738

**Published:** 2018-09-26

**Authors:** Xinyu Yang, Haijiang He, Jun Xu, Yikun Wei, Hua Zhang

**Affiliations:** 1 Faculty of Mechanical Engineering and Automation, Zhejiang Sci-Tech University, Hangzhou 310018, China; 2 Zhejiang Yilida Ventilator Company, Taizhou 318056, China; 3 State-Province Joint Engineering Lab of Fluid Transmission System Technology, Hangzhou 310018, China; 4 College of Energy Engineering, Zhejiang University, Hangzhou 310018, China

**Keywords:** entropy, Rayleigh–Taylor, turbulence, mixing, lattice Boltzmann method

## Abstract

Entropy generation rates in two-dimensional Rayleigh–Taylor (RT) turbulence mixing are investigated by numerical calculation. We mainly focus on the behavior of thermal entropy generation and viscous entropy generation of global quantities with time evolution in Rayleigh–Taylor turbulence mixing. Our results mainly indicate that, with time evolution, the intense viscous entropy generation rate $s_{u}$ and the intense thermal entropy generation rate $S_{\theta }$ occur in the large gradient of velocity and interfaces between hot and cold fluids in the RT mixing process. Furthermore, it is also noted that the mixed changing gradient of two quantities from the center of the region to both sides decrease as time evolves, and that the viscous entropy generation rate ${\langle S_{u}\rangle }_{V}$ and thermal entropy generation rate ${\langle S_{\theta }\rangle }_{V}$ constantly increase with time evolution; the thermal entropy generation rate ${\langle S_{\theta }\rangle }_{V}$ with time evolution always dominates in the entropy generation of the RT mixing region. It is further found that a “smooth” function ${\langle S_{u}\rangle }_{V}∼t^{1/2}$ and a linear function ${\langle S_{\theta }\rangle }_{V}∼t$ are achieved in the spatial averaging entropy generation of RT mixing process, respectively.

## 1. Introduction

Rayleigh–Taylor (RT) instability occurs in a large number of engineering applications. In general, it mainly originates at the interface between a heavy fluid and a light fluid due to a gravitational field [1,2,3,4,5,6]: the colder layer is placed above the hotter layer due to a gravitational field causing the accumulation of two layers in a single-phase fluid [3]. Zhou [4] studied the statistical properties of the kinetic energy dissipation rates and thermal energy dissipation rates in 2D RT turbulence. One of the critical issues is a deeper comprehension of the transport mechanisms of both the viscosity and thermal entropy generation rate inside the mixing zone during RT turbulence. The local entropy generation rates that play a substantial role in energy-loss are the viscosity and thermal entropy generation rate fields [7,8], which are given in two dimensions.

(1) $$S_{u}=\frac{\mu }{\theta }\left\{2\left[{\left(\frac{\partial u}{\partial x}\right)}^{2}+{\left(\frac{\partial v}{\partial y}\right)}^{2}\right]+{\left(\frac{\partial u}{\partial y}+\frac{\partial v}{\partial x}\right)}^{2}\right\}$$

And

(2) $$S_{\theta }=\frac{\kappa }{\theta^{2}}\left[{\left(\frac{\partial \theta }{\partial x}\right)}^{2}+{\left(\frac{\partial \theta }{\partial y}\right)}^{2}\right]$$

where $S_{u}$ and $S_{\theta }$ represent the direct viscosity and thermal entropy generation rate. They mainly measure the magnitudes of gradients of the temperature and velocity fields. Many aspects of entropy generation have been investigated in the past decades [9,10,11,12,13,14,15]. Qing (2016) [9], Abbas (2016) [10], Yang (2018) [11], Pizzolato (2016) [12], Mahian (2011) [13], Bhatt (2016) [14,15], etc. have discussed local entropy generation rates including a large amount of phenomenological information. Zahmatkesh et al. [16] found that the discontinuous heating/cooling boundary can bring about a high generation rate. Oztop et al. [17] reported the thermal entropy generation of a nanofluid in various magnetic field obstacles. Sciacovelli et al. [18] presented a review about entropy generation analysis in various engineering systems. Wei et al. [19] discovered that entropy generation in natural convection can be effected by different Prandtl numbers.t. Wang et al. [20] discovered that discrete heat boundary conditions can affect entropy generation in natural convection. Jin [21] reported that entropy is a powerful method by the tool of Computational Fluid Dynamics.

Based on the above discussions, the statistics of the viscosity and thermal entropy generation rates in two-dimensional RT turbulence by means of data obtained from the lattice Boltzmann method are investigated in this paper. As time evolution, viscous and thermal entropy generation in RT turbulence mixing vary to give brilliant physical characteristics. In this paper, we mainly focus on the statistics of viscous and thermal entropy generation in RT turbulence mixing with time evolution. The characteristic of local distributions about viscous and thermal entropy generation are analyzed with time evolution. Our results suggest that the values of the viscous entropy generation can be neglected compared to the growth rate of the total entropy generation rate of the system [19].

Numerical simulations of turbulent RT mixing are implemented by the double distribution lattice Boltzmann method (LBM). It is well-known that LBM possesses great potential to simulate single phase flow, gas-liquid phase flow, and heat transfer flow problems [22,23,24,25,26,27,28,29,30,31,32]. LBM not only encompasses their fully local stream-and-collide nature, but also possesses the potential advantage that the transfer of all information is local in time and space; additionally, the lattice Boltzmann equation is an effective approach to implement parallel computing.

In this paper, we firstly review the RT instability and entropy generation rate. In Section 2, the dynamics equations of thermal fluid and double distribution LBM are introduced in the present study. In Section 3, the temperature, viscosity, and thermal entropy generation rates with time evolution are given; some discussions are illustrated. Finally, conclusions are presented.

## 2. Macroscopic Dynamics Equation of Thermal Fluid and Lattice Boltzmann Method

### 2.1. Macroscopic Dynamics Equation of Thermal Fluid

The classical Oberbeck–Boussinesq equations are given by the following equations to study the thermal fluid dynamics equations [6,8].

(3) $$\frac{\partial \rho }{\partial t}+\nabla \cdot (\rho u)=0$$

(4) $$\frac{\partial (\rho u)}{\partial t}+\nabla \cdot (\rho uu)=-\nabla p+\nabla \cdot (2\rho \nu S)-g\beta \Delta \theta$$

(5) $$\frac{\partial \theta }{\partial t}+u\cdot \nabla \theta =\kappa \nabla^{2}\theta$$

where ν represents the kinematic viscosity coefficient, κ represents the coefficient of diffusivity, ρ represents the density of fluid, u represents the macroscopic velocity, p represents the fluid pressure, S is the shear stress, θ is the macroscopic temperature, and Δθ is the temperature difference, respectively.

### 2.2. Double Distribution Lattice Boltzmann Method

The lattice Boltzmann equation to simulate fluid flow is given as [29,30,31]:

(6) $$f_{i}(x+c_{i}\Delta t,t+\Delta t)=f_{i}(x,t)+(f_{i}^{eq}(x,t))-f_{i}(x,t))/\tau_{\nu }+F_{i}$$

Here $f_{i}(x,t)$ denotes the distribution function of density, $c_{i}$ is the discrete velocity. $F_{i}$ represents the discrete force term in Equation (6), $\tau_{\nu }$ is the relaxation time for density evolution equation in lattice Boltzmann equation, and $f_{i}^{eq}$ is the density distribution equilibrium function.

The lattice Boltzmann equation for the temperature field:

(7) $$g_{i}(x+c_{i}\Delta t,t+\Delta t)=g_{i}(x,t)+(g_{i}^{eq}(x,t))-g_{i}(x,t))/\tau_{\theta }$$

where $g_{i}(x,t)$ denotes the temperature distribution function, $\tau_{\theta }$ denotes the relaxation times for temperature evolution equation in the above equation and $g_{i}^{eq}$ is the temperature distribution equilibrium function. The density distribution equilibrium function and the temperature distribution equilibrium function are represented by Equations (8) and (9) [30], respectively.

(8) $$f_{i}^{eq}=\rho w_{i}[1+\frac{c_{i}\cdot u}{c_{s}^{2}}+\frac{{\left(c_{i}\cdot u\right)}^{2}}{c_{s}^{2}}-\frac{u^{2}}{2c_{s}^{2}}]$$

(9) $$g_{i}^{eq}=\theta w_{i}[1+\frac{c_{i}\cdot u}{c_{s}^{2}}+\frac{{\left(c_{i}\cdot u\right)}^{2}}{c_{s}^{2}}-\frac{u^{2}}{2c_{s}^{2}}]$$

where $w_{i}$ represents the weight coefficient [25]. The relations among kinematic viscosity ν and the coefficient of thermal diffusivity κ, and the relaxation time are given as:

(10) $$\nu =\frac{2\tau_{\nu }-1}{6}\frac{{\left(\Delta x\right)}^{2}}{\Delta t},\;\kappa =\frac{2\tau_{\theta }-1}{6}\frac{{\left(\Delta x\right)}^{2}}{\Delta t}$$

where Δt is the unit time and Δx is the unit space. The Macroscopic density, velocity, and temperature are represented by Equation (9).

(11) $$\rho =\sum_{i=0}^{8}f_{i}\;\;\;\rho u=\sum_{i=0}^{8}c_{i}f_{i}\;\;\;\theta =\sum_{i=0}^{8}g_{i}$$

The Mesoscopic equation for density, momentum (Equation (6)), and temperature (Equation (7)) are spread by a Chapman–Enskog expansion. The Macroscopic Oberbeck–Boussinesq equations (Equations (3)–(5)) are obtained by a macroscopic length scale ($x_{1}=\varepsilon x$) and two macroscopic time scales ($t_{1}=\varepsilon t$, $t_{2}=\varepsilon t$). As one spatial scale $\partial_{x}=\varepsilon \partial_{\alpha }$, and two time scales $\partial_{t}=\varepsilon \partial_{t_{1}}+\varepsilon^{2}\partial_{t_{2}}$ are implemented. The macroscopic classical Oberbeck–Boussinesq equations (Equations (3) and (4)) can be reproduced by the Chapman–Enskog expansion of executing the Equations (6) and (7). The *Rayleigh* number (*Ra*) is an important dimensionless parameter in the turbulent RT mixing flow. The expression of *Ra* in the simulation of LBM is given by:

(12) $$Ra=\beta \Delta \theta gH^{3}/\nu \kappa$$

where β is the coefficient of thermal conductivity, g represents the acceleration of gravity. The *Nusselt* number (*Nu*) is also one of most important dimensionless parameters in the turbulent RT mixing flow. The expression of *Nu* in the simulation of LBM is given by:

(13) $$Nu=1+\langle u_{y}\theta \rangle /\kappa \Delta \theta H$$

where Δθ represents the difference of temperature between the bottom boundary and the top boundary, H denotes the height of channel, $u_{y}$ is the vertical macroscopic velocity, and 〈.〉 is the average value of the entire computational domain.

In this paper, the nonequilibrium extrapolation method and the periodic condition are used. The expressions of nonequilibrium extrapolation method are given by [20]:

(14) $$f_{i}(x_{b},t)=f_{i}^{eq}(\rho_{w},u_{w})+(f_{i}(x_{f},t)-f_{i}^{eq}(\rho_{f},u_{f}))$$

(15) $$g_{i}(x_{b},t)=g_{i}^{eq}(\rho_{w},u_{w})+(g_{i}(x_{f},t)-g_{i}^{eq}(\rho_{f},u_{f}))\;$$

where the nonequilibrium contribution is derived from the fluid node $x_{{}_{f}}$ next to $x_{b}$ along the boundary normal vector [27]. The expressions of the periodic condition method are given by [27]:

(16) $$f_{i}(x,t)=f_{i}(x+L,t)$$

(17) $$g_{i}(x,t)=g_{i}(x+L,t)$$

where the vector **L** is the periodicity direction and the length of the flow pattern.

## 3. Some Numerical Results and Discussions

The uniform grid is implemented for all of the following numerical simulations. The convergence criterion is set for all cases. A clear scaling can be seen for *Nu*(*Ra*) for nearly four decades from Ra ≈ 10^6^ to 10^10^. The compensated plots in the insets give [4],

(18) $$Nu=Ra^{0.5}$$

The grid-dependence study of the results is implemented. One example of the Rayleigh number of Ra = 9.8 × 10^9^ is presented in Table 1. In this study, the number of grid points is taken as the same in both the x and y directions. That is, the grid size is taken as M×N, where *M* is the grid number in the transverse coordinates direction and N is the grid number in the longitudinal coordinates direction. The calculated Nusselt number changing with M×N, is presented in Table 1. From this table, it is clearly seen that when M×N increases, the calculated *Nusselt* number quickly approaches the benchmark result. When the grid size further increases from 2056 × 4112 to 2200 × 4400, there is not much improvement in the result. So one can say that for Ra = 9.8 × 10^9^, the grid size of 2056 × 4112 can give very accurate results. As shown in Table 1, one can see that result of LBM for the relation of Nu(Ra) is well consistent with theoretical value of Nu(Ra) [4].

**Figure 1:**
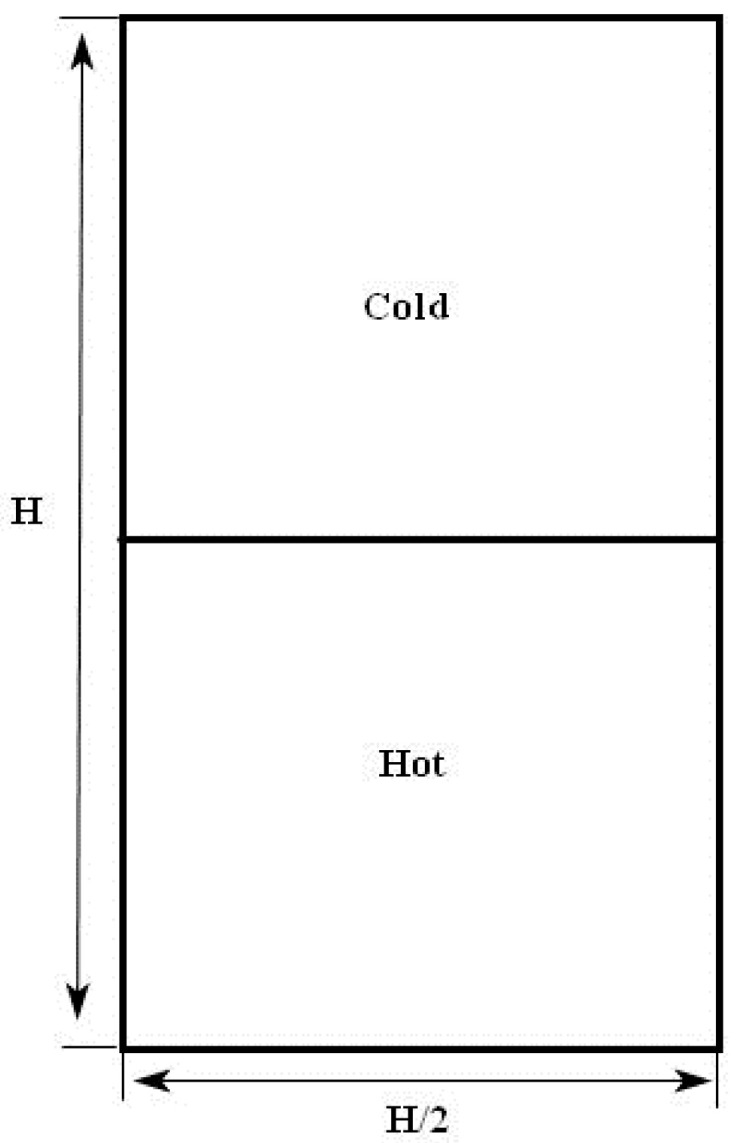
Computational schematic geometry.

To ensure adequate resolution for $S_{u}$ and $S_{\theta }$, 2056 × 4112 lattices were implemented using the double distribution LBM of the present work. In the initial stage, the system is at rest. In the upper half of the calculation area, the fluid is cold. The fluid is hot in the lower half of the calculation area. If y is greater than H/2, the temperature equals −0.5 in Figure 1 and the temperature equals to 0.5 when y is less than *H*/2. An initial temperature, $\theta_{0}$, is executed in the colder uniform fluid layer and placed on top of the hotter one. To achieve the repeatability of whole flow field, a total of eight independent realizations in RT evolution were performed by giving some perturbed interfaces. In all the simulations, *Ag* = 0.25, Ra = 9.8 × 10^9^, and the corresponding Prandtl number is Pr=v/κ=7. For the vertical boundaries, periodic boundary conditions are executed. The no-slip boundary conditions are adopted in the top and bottom boundary conditions. It is noted that in the previous studies, Zhou et al. [4] investigated the statistical properties of kinetic and thermal energy dissipation rates in RT turbulence mixing. Here, some new analysis investigating the viscous and thermal entropy generation are performed in RT turbulence mixing.

### 3.1. Analysis of Flow and Temperature Field

**Figure 2:**
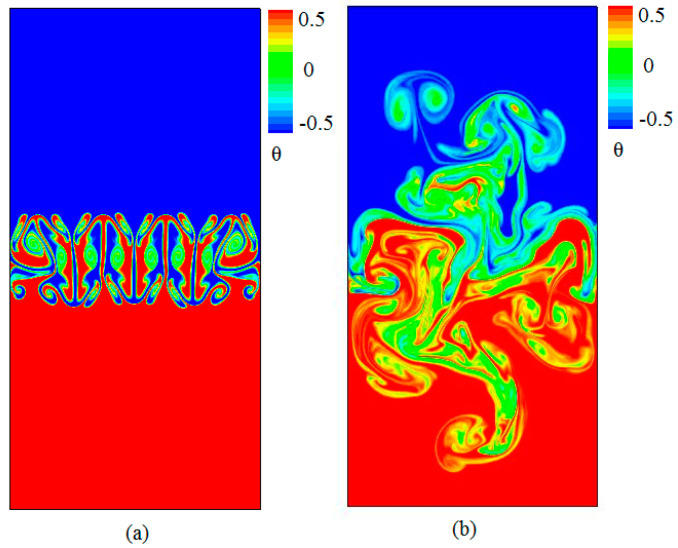
Snapshots of the temperature fields with time evolution obtained at times (**a**) t/τ=1.2, and (**b**) t/τ=4.

Figure 2 shows that the snapshots of the temperature fields with time evolution obtained at times (a) t/τ=1.2, and (b) t/τ=4, where $\tau =\sqrt{H/Ag}$ is the characteristic time in the time evolution of RT mixing. It is clearly seen that the flow is dominated by a large number of plumes and spikes (large-scale structures) in the turbulence regime. The hot fluid rises up as small plumes or spikes while the cold fluid falls down as slender spikes. The cold fluid and the hot fluid gradually become mixed with time evolution. A mixed zone of the cold fluid and the hot fluid develops and grows with time evolution. Finally, large-scale structures appear which is consistent with the previous studies [4]. In the present work, special attention is paid to the statistical properties of $S_{u}$ and $S_{\theta }$ within this range.

### 3.2. Analysis of $S_{u}$ and $S_{\theta }$ in RT Turbulence Mixing

**Figure 3:**
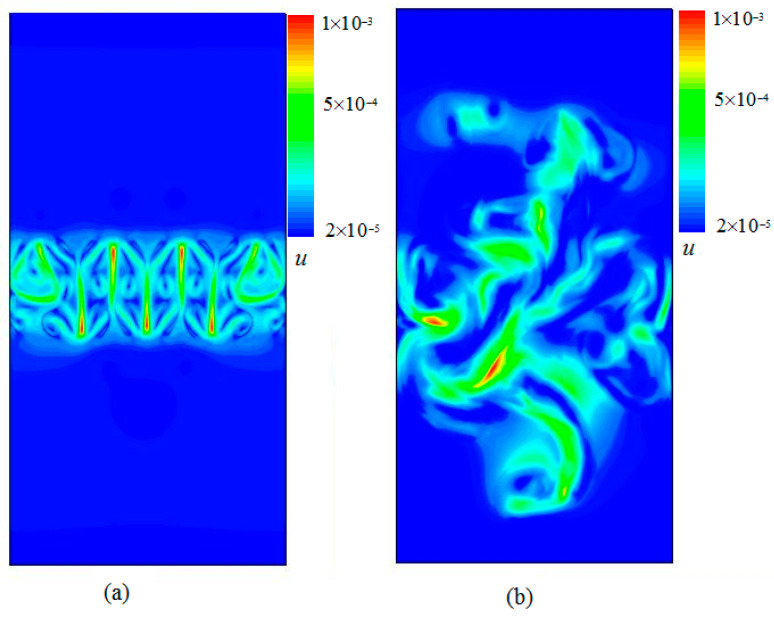
Snapshots of the velocity fields with time evolution obtained at times (**a**) t/τ=1.2, and (**b**) t/τ=4.

**Figure 4:**
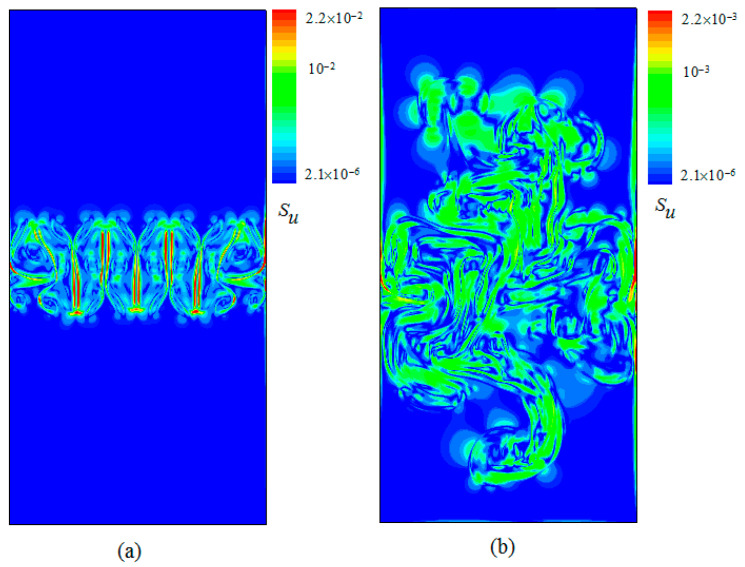
Snapshots of the viscous entropy generation with time evolution obtained at times (**a**) t/τ=1.2, and (**b**) t/τ=4.

Figure 3 represents the distribution of constant velocity contours with time evolution obtained at times (a) t/τ=1.2 and t/τ=4. Figure 4 displays the snapshots of the viscous entropy generation with time evolution obtained at times t/τ=1.2 and t/τ=4. As shown in Figure 2 and Figure 3, one clearly sees that the viscous entropy generation rate ($S_{u}$) and the velocity with time evolution always occur in the RT mixing region, which indicates that the loss of flow is also mainly concentrated in this mixing area. It is also further seen that the intense $S_{u}$ usually concentrates on the steepest velocity gradient in the mixing process, which is consistent with the viscous entropy generation in Rayleigh–Bénard convection [19].

**Figure 5:**
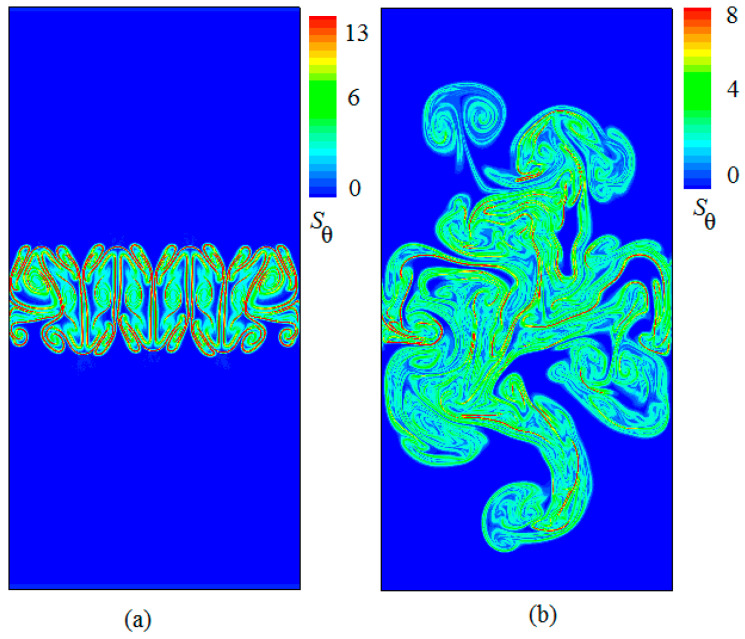
Snapshots of the thermal entropy generation with time evolution obtained at times (**a**) t/τ=1.2, and (**b**) t/τ=4.

Figure 5 displays the snapshots of thermal entropy generation with time evolution obtained at times (a) t/τ=1.2 and (b) t/τ=4. As shown in Figure 4, one clearly sees that with time evolution the $S_{\theta }$ always occurs in the RT mixing region. Further, it was found that as the time evolution progressed, the intense $S_{\theta }$ focuses on the interfaces between the hot and cold fluids in the RT mixing process, which is also consistent with the thermal entropy generation in Rayleigh–Bénard convection [19].

**Figure 6:**
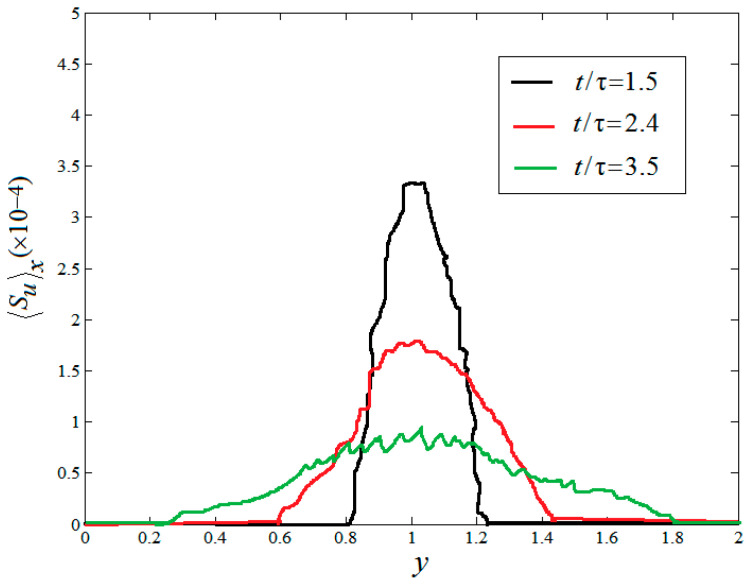
Mean vertical profiles of the horizontal root-mean-square (rms) viscous entropy generation.

**Figure 7:**
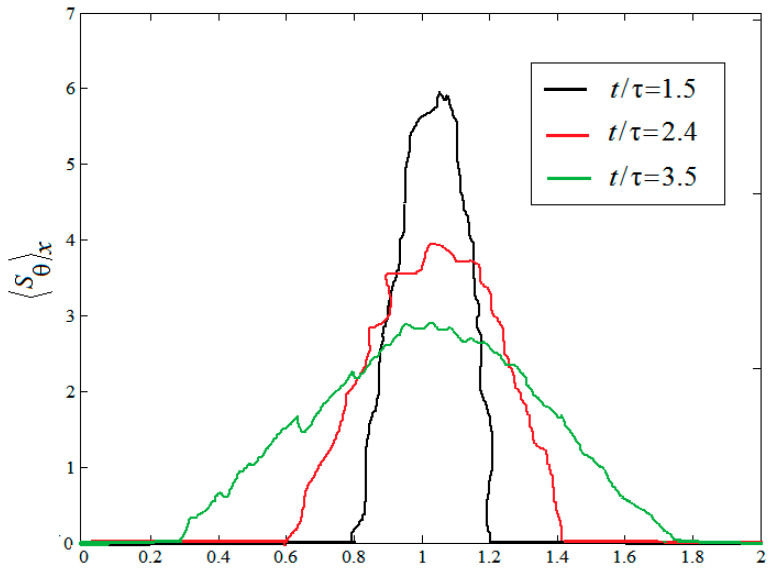
Mean vertical profiles of the horizontal rms thermal entropy generation.

In the above section, the various instantaneous viscous entropy generation rates and thermal entropy generation rates are presented in the field of space. In the following section, the mean values of the viscous entropy generation rate and thermal entropy generation rates are analyzed in space. Figure 6 and Figure 7 display the temporal evolution of the mean vertical profiles of the horizontal and vertical root-mean-square (rms) viscous entropy generation ${\langle S_{u}\rangle }_{X}$ and the thermal entropy generation ${\langle S_{\theta }\rangle }_{X}$ at times t/τ=1.5, t/τ=2.4 and t/τ=3.5, respectively, where $i_{rms}=\sqrt{{\langle {\left(i-{\left(i\right)}_{j}\right)}^{2}\rangle }_{j}}$ is the RMS value of i with $i=S_{u}$, $S_{\theta }$ and with j=x for a horizontal average. As shown in Figure 5 and Figure 6, one can see that all profiles of ${\langle S_{u}\rangle }_{X}$ and ${\langle S_{\theta }\rangle }_{X}$ display a similar shape, not far from a parabola at the temporal evolution. However, the behaviors of the amplitudes of two quantities vary as time evolves. It is also found that the mixed changing gradient of two quantities from the center of the region to both sides decrease as time evolves.

**Figure 8:**
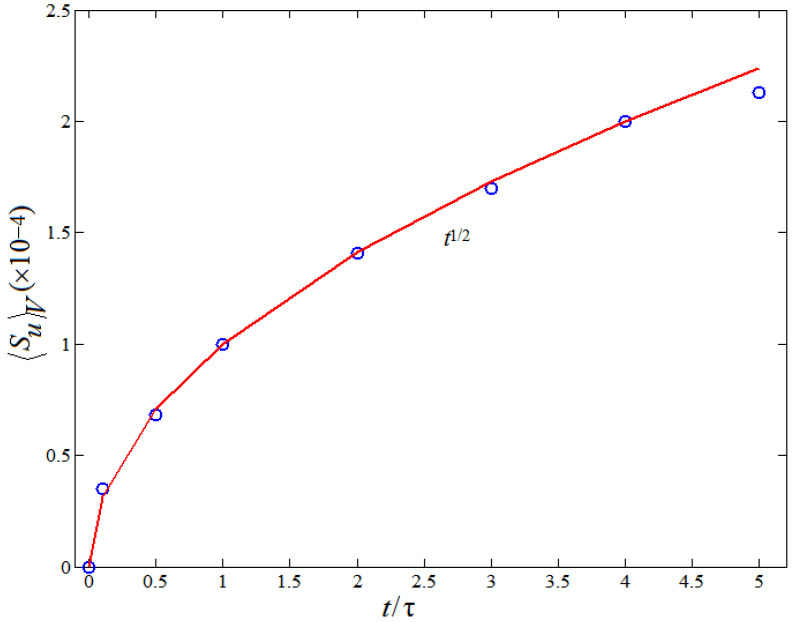
Temporal evolution of the viscous entropy generation rate normalized by the computational grid spacing.

Figure 8 shows the time behaviors of the viscous entropy generation rate ${\langle S_{u}\rangle }_{V}$ normalized by the computational grid spacing. From Figure 8, it is clearly seen that the viscous entropy generation rate ${\langle S_{u}\rangle }_{V}$ always increases with time evolution. The solid line represents the theoretical prediction fitted approximately by the least square method according to the numerical results of LBM in Figure 8. A “smooth” function ${\langle S_{u}\rangle }_{V}∼t^{1/2}$ is approximately achieved.

**Figure 9:**
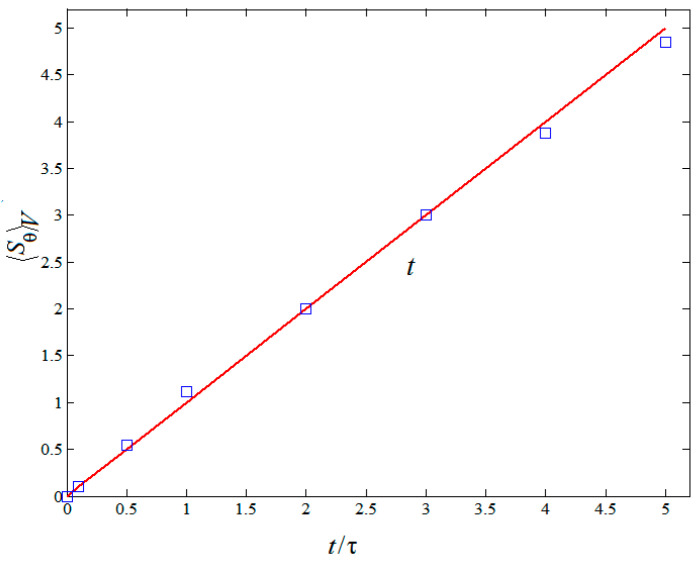
Temporal evolution of the thermal entropy generation rate normalized by the computational grid spacing.

The time behaviors of thermal entropy generation rate ${\langle S_{\theta }\rangle }_{V}$ normalized by the computational grid spacing in RT mixing are plotted in Figure 9. As shown in Figure 9, one can clearly see that the thermal entropy generation rate ${\langle S_{\theta }\rangle }_{V}$ increases with time evolution. The solid line in Figure 9 represents the theoretical prediction fitted approximately by the least square method according to the numerical results of LBM. The linear function ${\langle S_{\theta }\rangle }_{V}∼t$ was approximately obtained. Comparing Figure 7 with Figure 8, it is seen that ${\langle S_{\theta }\rangle }_{V}$ is almost four orders of magnitude greater than ${\langle S_{u}\rangle }_{V}$ in turbulent RT mixing. It is further indicated that the thermal entropy generation rate with time evolution plays a main role in the entropy generation of RT mixing.

## 4. Conclusions

In this paper, entropy generation rates in two-dimensional Rayleigh–Taylor turbulence mixing with time evolution are investigated. The various instantaneous viscous entropy generation rates and thermal entropy generation rates were studied in the field of space. Mean values of the viscous entropy generation rate and thermal entropy generation rate were also discussed in space. Several major findings are summarized.

First of all, it is shown that the intense viscous entropy generation rate $S_{u}$ with time evolution always focuses on the large gradient of velocity in the RT mixing region. With progressive time evolution, the intense thermal entropy generation rate $S_{\theta }$ also focuses on the interfaces between the hot and cold fluids in the RT mixing process. In addition, all profiles of ${\langle S_{u}\rangle }_{X}$ (the mean vertical profiles of the horizontal and vertical root-mean-square) and ${\langle S_{\theta }\rangle }_{X}$ possess a similar shape, not far from a parabola at the temporal evolution. The mixed changing gradient of two quantities from the center of the region to both sides decrease as time evolves. One can also obtain that the viscous entropy generation rate ${\langle S_{u}\rangle }_{V}$ and the thermal entropy generation rate ${\langle S_{\theta }\rangle }_{V}$ constantly increase with time evolution. A “smooth” function ${\langle S_{u}\rangle }_{V}∼t^{1/2}$ and a linear function ${\langle S_{\theta }\rangle }_{V}∼t$ are achieved, respectively. Furthermore, it is found that the thermal entropy generation rate ${\langle S_{\theta }\rangle }_{V}$ with time evolution always plays a main role in the entropy generation of RT mixing region.

## Figures and Tables

**Table 1:** Grid-dependence study for Rayleigh–Taylor (RT) turbulence mixing at Ra = 9.8 × 10^9^.

Mesh	500 × 1000	1000 × 2000	2056 × 4112	2200 × 4400	(*Nu-Ra*) Theoretical Value [4]
*Nu*	96,573.33	98,089.26	98,993.76	98,993.75	98,994.95
